# Phage display as a promising approach for vaccine development

**DOI:** 10.1186/s12929-016-0285-9

**Published:** 2016-09-29

**Authors:** Leili Aghebati-Maleki, Babak Bakhshinejad, Behzad Baradaran, Morteza Motallebnezhad, Ali Aghebati-Maleki, Hamid Nickho, Mehdi Yousefi, Jafar Majidi

**Affiliations:** 1 Immunology Research Center, Tabriz University of Medical sciences, Tabriz, Iran; 2 Drug Applied Research Center, Tabriz University of Medical Sciences, Tabriz, Iran; 3 Department of Immunology, Faculty of Medicine, Tabriz University of Medical Sciences, Tabriz, Iran; 4 Department of Genetics, Faculty of Biological Sciences, Tarbiat Modares University, Tehran, Iran

**Keywords:** Bacteriophage, Phage display, Peptide library, Vaccine development, Biopanning, Mimotope

## Abstract

Bacteriophages are specific antagonists to bacterial hosts. These viral entities have attracted growing interest as optimal vaccine delivery vehicles. Phages are well-matched for vaccine design due to being highly stable under harsh environmental conditions, simple and inexpensive large scale production, and potent adjuvant capacities. Phage vaccines have efficient immunostimulatory effects and present a high safety profile because these viruses have made a constant relationship with the mammalian body during a long-standing evolutionary period. The birth of phage display technology has been a turning point in the development of phage-based vaccines. Phage display vaccines are made by expressing multiple copies of an antigen on the surface of immunogenic phage particles, thereby eliciting a powerful and effective immune response. Also, the ability to produce combinatorial peptide libraries with a highly diverse pool of randomized ligands has transformed phage display into a straightforward, versatile and high throughput screening methodology for the identification of potential vaccine candidates against different diseases in particular microbial infections. These libraries can be conveniently screened through an affinity selection-based strategy called biopanning against a wide variety of targets for the selection of mimotopes with high antigenicity and immunogenicity. Also, they can be panned against the antiserum of convalescent individuals to recognize novel peptidomimetics of pathogen-related epitopes. Phage display has represented enormous promise for finding new strategies of vaccine discovery and production and current breakthroughs promise a brilliant future for the development of different phage-based vaccine platforms.

## Background

Viruses are biochemical complexes made up of genomic and proteomic materials. All of the physical, chemical, and biological characteristics associated with the structure and function of these biological entities are specified by their genetic sequences. Viruses infect their hosts through recognizing specific receptors on the surface of host cells and subsequently transfer their genetic information into the cells. The delivered viral genome hijacks the host metabolism in order to produce more virions, thereby propagating the viral population [1]. Within the recent decades, a considerable body of data has accumulated with regard to the molecular basis of structural and functional features of viruses. These findings have led to a paradigm shift in our understanding of viruses from disease-causing pathogens to health-giving biomedical tools.

The discovery of bacteriophages caused a surpsrisingly dramatic increase in the exploitation of viruses in various scientific and research territories. Bacteriophages, or more colloquially phages, are viruses that infect bacterial cells. These prokaryotic viruses are known as the most successful life forms on earth because they have abundance and genetic diversity higher than any other organism. The number of phages in nature is estimated to be 10^31^particles. Nevertheless, recent metagenomic studies have revealed that about two thirds of gene sequences identified in phages isolated from environmental samples are novel with no homologous equivalents in databases [2, 3]. Therefore, it seems our current knowledge of phage diversity represents only the tip of the iceberg. Phage history was initiated a century ago when Felix d’Herelle discovered the power of these viral agents to cure pathogenic bacterial infections. This therapeutic modality, called phage therapy, waned with the rise of antibiotic era. However, phages continued to serve as favorable model organisms and experimental systems for the development of a broad array of molecular tools that contributed to decode many fundamental mysteries in biology [4]. Numerous lines of progress inspired by phage research laid the foundation for a new discipline now known as molecular biology. Nowadays, intractable problems triggered by the ever-increasing number of antibiotic-resistant bacterial species have driven a renaissance of interest in the decades-long-neglected field of phage therapy.

Phage research represents a dynamically developing field and is continually expanding in scope. During its one-century-long history, phage study has witnessed ongoing breakthroughs providing new perspectives to the biomedical applications of these viral agents. Currently, phage applications cover a wide variety of areas including studies of protein-protein interactions [5], antiviral research [6, 7], anticancer research [8], biosensor design [9, 10], development of targeted gene/drug delivery platforms [11–13], and nanotechnology [14, 15], just to mention a few. Vaccine development is one of the most important applications of phage display receiving huge attention in recent years. In the current article, we discuss the potential of phages as vaccine delivery vehicles. Phages can be exploited for vaccine delivery in two formats of phage DNA and phage display vaccines. However, the largest contribution of phages to vaccine design arises from phage display technology. Therefore, our main focus will be on phage display methodology and its application for vaccine design. In this regard, high throughtput screening of phage combinatorial libraries through affinity selection for mimotope identification is particularly addressed.

## Phage display: epitopes on surface

**Fig. 1:**
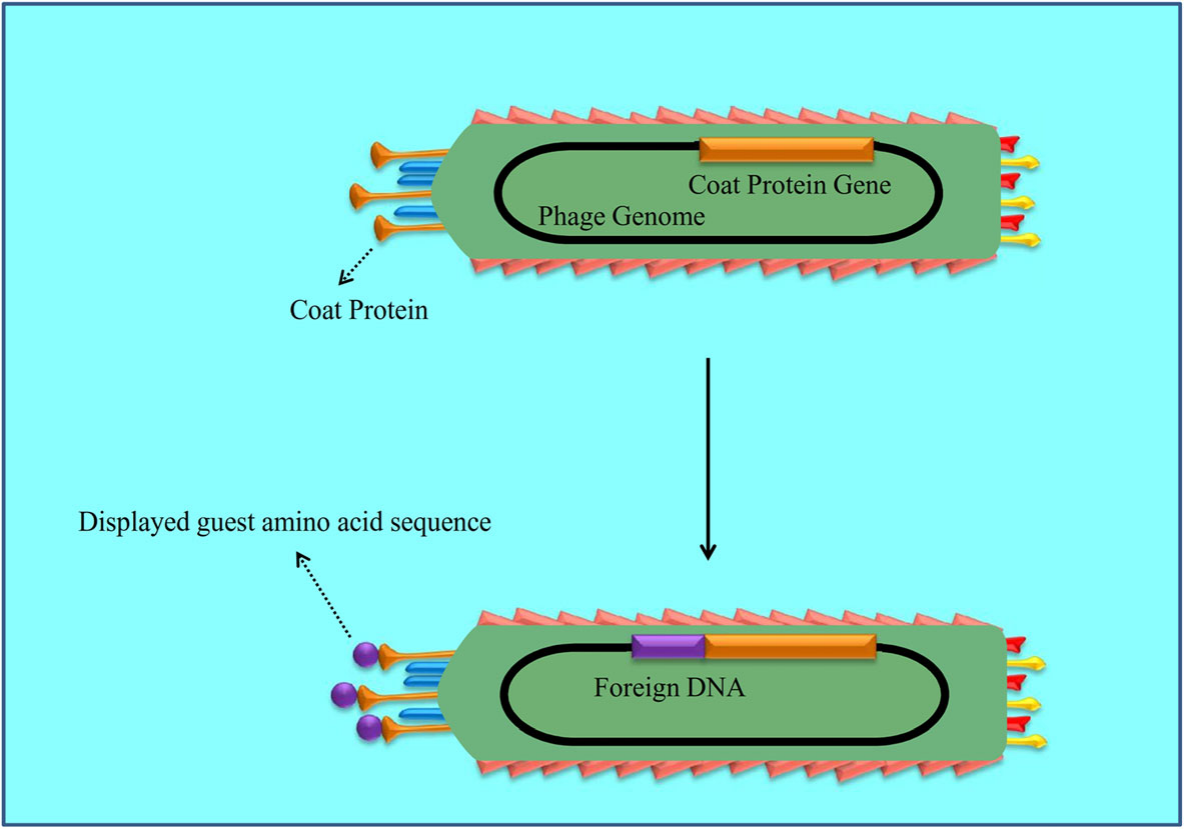
Schematic representation of phage display Cloning of foreign DNA into the gene which encodes one of the phage coat proteins leads to the expression of guest amino acid sequence as part of the relevant coat protein on the phage surface. In phage display, the displayed peptide (phenotype) and its encoding nucleotide sequence (genotype) are physically linked together in an individual phage particle

The introduction of phage display technology may be considered as a turning point in the history of phage research. Conceptually, phage display adapts the basic principles of genetic engineering and combinatorial chemistry. The launch of this technique is marked by the work of Smith and his colleagues in 1985 in which they first described the display of foreign polypeptides on the surface of filamentous phage particles [16]. In phage display, exogenous DNA is inserted into a specific site in the nucleotide sequence encoding one of the phage coat proteins. When phage particles infect their bacterial host and express their genes, guest amino acids encoded by foreign DNA are expressed as a part of the relevant coat protein. As a consequence, a fusion protein is displayed on the exposed surface of phage virion (Fig. 1). Guest residues in such fusion proteins are able to interact with a wide variety of external target molecules. The power of phage display technology results from its ability to establish a physical connection between phenotype (displayed peptide) and genotype (DNA sequence encoding the displayed peptide). This phenotype-genotype link forms the cornerstone of phage display methodology and makes it possible for researchers to isolate target-avid ligands displayed on the phage.

One of the hallmark characteristics of phage display technology is the capacity to produce very large libraries of peptides [17, 18]. This innovative development has turned phage display into a fast and reliable high throughput screening methodology. Therefore, many useful functional peptides with desired characteristics can be selected in a straightforward and effective manner. To construct a phage display library, a highly diverse pool of randomized oligonucleotide sequences are spliced into one of the phage coat proteins. By cloning a large quantity of oligonucleotide fragments into the phage genome, phage library may harbor millions or sometimes billions of unique and distinctive displayed peptide ligands. In fact, each phage virion of the library displays an individual type of guest peptide on its surface; however, the entire library contains a huge number of peptides. Since each virion can act as an independent vector and is capable of infecting bacteria, each phage particle selected from the library with its displayed peptide can be grown and propagated separately in host cells. Phage display libraries exhibit several benefits compared with other types of screening systems. Phage particles are rather small and it is estimated that 10^12^ particles may be present in 1 mL of solution [19]. The small size of phage virions allows researchers to screen a larger number of different particles in each round. Accordingly, a higher number of viral particles are checked to obtain the most promising binders with the strongest affinity to the target. Easy manipulation, safety, low cost of propagation, and access to numerous commercially available kits are other advantages associated with phage display library for screening purposes. Furthermore, one of the most important factors contributing to the widespread application of phage display is the convenience by which these libraries can be screened to select target-specific binders.

In the context of vaccine development, peptide-based phage display libraries fall into two main categories of natural peptide libraries (NPLs) and random peptide libraries (RPLs). For the establishment of NPLs, randomly fragmented DNA derived from the genetic material of an organism of interest is incorporated into the phage genome. This procedure can be applied to the fragments obtained from genomes of pathogenic microbes such as viruses, bacteria, etc. Thus, phage particles in NPLs display on their surface fragments of natural proteins. But, RPLs are constructed by cloning synthetic random degenerate oligonucleotide inserts into the phage genome. Compared with RPLs, NPLs are more likely to mount an antibody response that crossreacts with the native intact pathogen [20]. However, NPLs suffer from a major limitation in that the vast majority of clones in these libraries are nonfunctional. By contrast, RPLs have a universal nature and as a result of their construction mode even peptides not found within the antigen or intact pathogen can be displayed on the surface of library phages [21]. These libraries extend markedly the range of epitopes that can be displayed on phage virions. Currently, random peptide libraries are known as the most frequently used type of phage display libraries and have been used extensively to identify antigenic epitopes.

## Biopanning for screening of combinatorial phage display libraries

**Fig. 2:**
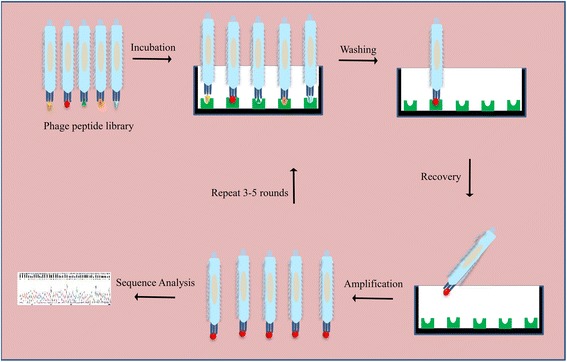
Biopanning for the identification of target-specific phage-displayed peptides The incubation of a randomized peptide phage display library with the target is followed sequentially by multiple washing steps (to remove the unbound phages), recovery of target-bound phages, and their amplification through bacterial infection. These steps are repeated 3–5 rounds. Finally, selected phage clones displaying peptides with the highest affinity towards the target are identified through DNA sequencing

Since their birth, phage display libraries have been screened against a wide variety of targets. At first, purified biomolecules (like a receptor, an antibody, etc) were used as the target. However, in subsequent years phage display selection also served to isolate peptides that could bind to complex biological targets. In this context, whole cells in culture and living animals were exploited as the target. Therefore, ligands were obtained that could bind to cell surface receptors or home to the vasculature of specific tissues [22, 23]. Even non-biological elements such as semiconductors have also found application as screening targets for phage display libraries. The screening of phage display libraries for the enrichment of peptides that specifically bind to the target of interest is performed through an affinity selection-based strategy called biopanning (Fig. 2). In biopanning, a population of phage particles (including a library of random peptides) is incubated with the target for a specified period of time. Then, the target is exposed to a series of stringent washing steps. Phages whose displayed peptides bind to the target are captured, whereas the unbound or weakly-bound phages are removed. The interacting phages are then eluted by utilizing a strategy that breaks chemical bonds between the target and the displayed peptide without compromising phage infectivity. Altering pH, adding a competing ligand, applying a denaturing agent or using a proteolytic enzyme are approaches commonly exploited for the release and retrieval of the target-bound phages [24]. These methods can be used for virion recovery because phage particles are extremely stable under harsh conditions. The recovered phages are then propagated by infecting bacterial host cells. Although the above-mentioned steps can enrich phage particles with high affinity for the target, the library is still relatively heterogeneous. As a result, the amplified pool of target binders needs to be exposed to the target for another several rounds. Typically, three to five rounds of affinity selection suffice to reach the most-strongly binding ligands. Finally, target-specific binders are identified through DNA sequencing of the selected phages. During rounds of biopanning, the stringency of selection can be intensified by progressively increasing the number or duration of washes in order to isolate phage clones with the highest affinity towards the target. Biopanning procedure can be undertaken in different formats. The most straightforward method of biopanning is immobilization of the target on a solid support such as multi-well microtiter plates, polystyrene tubes, nitrocellulose surfaces, and monolithic columns [25]. Immobilization on solid phase may be achieved either by direct attachment of the target to the surface of solid support or by biotinylating the target and then immobilizing it on a streptavidin-coated surface.. In the last several years, a novel type of phage display screening on solid surface has been developed. In this selection scheme, a rationally designed peptide array library is synthesized on functionalized cellulose membrane by a fully automated robotic system. The peptide library is then incubated with fluorescently labeled cells. Here, cell fluorescence is used to detect peptide-target cell interactions. This peptide array whole cell binding assay has been used successfully to isolate peptides that bind specifically to breast cancer cells. However, this methodology cannot be used independently and is complementary to conventional phage display for further optimization of the originally selected target cell-binding peptides [26]. As an alternative to target immobilization on a solid support, biopanning can also be carried out in solution phase. In this screening procedure, the library can be reacted with the target in solution and then the phage-target complexes are affinity captured onto an affinity matrix specific for the target. Some sort of affinity tag on the target is needed for affinity capture to occur [27]. For example, if the target is a protein with glutathione S-transferase (GST), maltose-binding protein (MBP) or polyhistidine affinity tag, the phage-target complex can be captured on glutathione, amylose, or nickel beads, respectively. Also, protein A and protein G beads can be applied to the capture of antibody targets. Solution biopanning offers some advantages over solid phase screening including enhanced accessibility of the target-associated ligand binding sites to the library, avoiding partial denaturation of the target on the solid plastic surface, and requiring an extremely less number of target molecules for each experiment. Recently, a new approach has been used for the affinity selection of phage-displayed peptides that bind to soluble protein targets. In this method, a random peptide library is incubated with the target protein affinity captured onto protein G magnetic beads followed by transfer of phage-magnetic bead conjugates to the organic solution. The removal of unbound phages and recovery of target-bound phages are performed through centrifugation. This methodology that applies the concept of organic phase separation to the affinity selection of phage display libraries is called magnetic beads via organic phase separation (MOPS) [28]. MOPS has been demonstrated to present high efficacy in selecting binders to Fc-fusion constructs and improve the isolation of specific binders for soluble protein targets that could not be enriched by conventional biopanning. In general, the exploitation of paramagnetic beads for target immobilization brings some benefits for phage display library screening that include enhanced surface area for binding, improved convenience and thoroughness of washing steps, and the appropriate orientation of binding domains in the target molecule. Another interesting approach for biopanning is using magnetic nanoparticles. In this strategy, biopanning can be accomplished through conjugating the target molecule onto the surface of magnetic nanoparticles. In this condition, magnetic force may be applied to separate the phage-target complex. Although the process of target antigen conjugation is tedious and time-consuming, the ability to take advantage of different chemical and enzymatic conjugation methods has made it easier to use semi-automated biopanning platforms [25]. Semi-automated magnetic biopanning based on magnetic nanoparticles allows the target to bind effectively to the surface of nanoparticles and enhances the accessibility of the target to phage particles.

In case of in vivo panning, selection procedure is taken to a new level of sophistication. In this screening methodology, peptides are selected only if they can overcome various inherent biological barriers present in in vivo environments (e.g. stability in the serum, cellular access, etc) and reach their targets. This in vivo approach has proven largely suceesful in the discovery of numerous tissue-specific homing peptides [29–31]. It can also be applied to identify peptides that home to a variety of cancer tissues [32–34]. For in vivo panning, phage library is administered intravenously (usually through the tail vein) into a mouse and allowed to circulate for a certain time period. The animal is then sacrificed, the desired organs or tissues are then extracted, and homogenized in saline solution. Washing to remove the unbound or nonspecifically-bound phage clones is typically mediated via perfusion of the left ventricle. The organ- or tissue-associated phages are amplified through bacterial infection and injected into another animal. After several rounds, targeting peptide motifs for specific organs are identified. Although phages of the library tend to be distributed nonspecifically throughout the entire animal body, some phages with specific peptides displayed on their surface home to particular tissues.

After ligands are selected in vitro or in vivo, their binding to the target shoud be checked in terms of specificity. To validate the phage specificity and selectivity in binding to the target in vitro and in vivo, a variety of methods can be used such as immunostaining with anti-phage antibodies through immunocytochemistry (ICC) and immunohistochemistry (IHC) techniques, cellular enzyme-linked immunosorbent assay (ELSA), competitive assay by using free synthetic peptides, in vivo imaging via fluorescent or radioactive labeling of the selected phages or their synthetically produced displayed peptides, etc.

## Phage display: development of therapeutic antibodies

Phage display has been introduced as an efficient alternative to hybridoma technology for the generation of therapeutically relevant antibodies. Antibody phage display (APD) provides the opportunity to design and construct large libraries of antibody fragments. The expression of antibodies on the surface of phage particles represents a powerful platform for the isolation of monoclonal antibodies (mAbs) with desired binding characteristics that can serve for the diagnosis and immunotherapy of various disorders especially infectious diseases [35].

According to the source of antibody-encoding sequences, phage antibody libraries (PALs) are divided into two main types of natural and synthetic libraries. Natural PALs consist of rearranged V gene repertoires of immune or non-immune (naïve) donors. This type of antibody library has been constructed from different species including human, non-human primates, mouse, chicken, sheep, camel, and cattle. Immune libraries are considered as the most useful type of PALs for infectious diseases. They are constructed from immunized donors and typically exploited to obtain antibodies against a particular target antigen, e.g. an infectious viral or bacterial agent. A great advantage of immune PALs is that V genes harbor hypermutations and are affinity matured, though the toxicity of some antigens and ethical issues make it impossible to immunize human subjects with toxic, immunosuppressive, and life-threatening antigens. If immunization is not possible or is ethically impracticable, the construction of non-immune or naïve PALs is an alternative. These libraries are generated from rearranged V gene repertoires of B cells derived from healthy and non-immunized donors. Accordingly, ethical issues with regard to the active immunization of humans and animals are avoided. Also, specific antibodies against non-immunogenic and toxic antigens can also be obtained. However, the isolated antibodies generally show low affinity. This emphasizes the need to improve binding affinity of the selected antibodies by using affinity maturation in vitro [36, 37]. Unlike natural PALs that represent the naturally occurring diversity in the antibody repertoire, synthetic PALs harbor artificial diversity in V gene segments. In these PALs, V genes are reconstructed in vitro partially (semi-synthetic PALs) or completely (fully synthetic PALs). The in vitro reconstruction of V genes is made via randomization of complementarity determining regions (CDRs) [36]. The source of V genes for introducing diversity into semi-synthetic PALs might be un-rearranged germline V genes, rearranged V genes from immune and/or naïve donors and single or multiple framework scaffolds of antibodies. The randomization of CDRs in particular VH-CDR3 is frequently used for the construction of semi-synthetic PALs. VH-CDR3 is known as the most diverse loop among six CDRs of human antibodies in terms of both length and sequence. Fully synthetic PALs are constructed by using human antibody frameworks with randomized CDR cassettes. The production of these libraries is based on *in silico* design and de novo synthesis. Human Combinatorial Antibody Library (HuCAL) is an inspiring example of a fully synthetic PAL that was generated by analysis of sequence and structure of frameworks and CDR loop regions [38]. In this library, nucleotide randomization was introduced into the VH and VL-CDR3 regions of different synthetic master frameworks. Fully synthetic PALs are currently being investigated for further improvement in order to achieve antibodies with favorable clinical properties. These adjustments aim to optimize synthetic binding sites with finely tuned affinity, size, and valency, as well as to minimize the number of T cell epitopes.

The large size of full-length mAbs appears as a major challenge to achieve the desirable clinical outcomes. To circumvent these limitations of full-sized mAbs, smaller antibody formats with improved pharmacokinetic and pharmacodynamic properties have been developed including fragment antigen binding (Fab), single-chain variable fragment (scFv), and single domain antibody (sdAb). Interestingly, a novel category of antibody fragments called nanobdies is currently known as the smallest recombinant antigen binding domain (<15 kDa) with full functionality that can be produced. The emergence of nanobodies goes back to two decades ago. In the early 1990s, it was discovered serendipitously that around fifty percent of the humoral immune response of the Camelidae family is provided by a unique repertoire of fully functional antibodies that contain only heavy chain [39]. These antibodies, known as Heavy Chain Antibody (HCAb), are in striking contrast to the well-established structure of IgG in mammals that are composed of two identical heavy chains and two identical light chains. Nanobody or VHH is the variable domain of HCAbs and is responsible for their antigen binding feature. Nanobodies have received growing interest as a promising class of recombinant clinically valuable antibody fragments [40]. Compared with conventional antibodies, nanobodies have a more hydrophilic structure leading to their high solubility. Also, convex surface and long CDRs enable them to recognize epitopes that are cryptic and inaccessible for conventional antibody fragments (e.g. epitopes in the catalytic sites of enzymes). Due to the easy molecular manipulation, nanobodies are excellent for the production of multivalent antigen binding constructs [41]. As nanobodies are closely related to the human VH sequences, they show very low immunogenic potential. Nanobodies have served against various infectious agents for immunodiagnostic and immunotherapeutic purposes. Consistent with this, nanobodies have been demonstrated to represent potential application as an *anti-trypanosoma* agent. *Trypanosoma* is a parasite that escapes the host immune system via exposing the hypervariable epitopes of its variant surface glycoprotein (VSG), while the conserved epitopes of VSG are cryptic with less immunogenic potential. The immunization of *Camelus dromedarius* with the antigen VSG has led to the identification of a nanobody that is capable of targeting the conserved Asn-linked carbohydrate of VSG [42]. Also, the VSG-specific nanobody conjugated with β-lactamase has been exhibited to present capacity as a diagnostic tool for imunodetection of *Trypanasoma*. Furthermore, the conjugation of VGS-specific nanobody with truncated apolipoprotein L-1 that is able to lyse both sensitive and resistant *Trypanosoma brucei rhodesiense* has caused complete elimination of the parasite during acute and chronic phases of challenge infection in mouse models [43]. Several nanobodies have also been selected against the cell surface protein of the fungus *Malassezia furfur* [44]. These nanobodies are highly stable under harsh environmental conditions of shampoo formulation and can be used for inhibiting the growth of the fungus on the scalp or as a fungus-targeting molecule for the development of anti-dandruff drugs. Moreover, nanobodies against rotavirus isolated in the stomach acidic environment have indicated considerable reduction in the occurrence of rotavirus-induced diarrhea in mouse models [45]. In another line of research, nanobodies have been demonstrated to be efficient immunodiagnostic and immunotherapeutic agents against bacterial toxins. A nanobody with specific binding to the lipopolysaccharide (LPS) of *Neisseriameningitidis* has inhibited LPS attachment to human monocytes, thus interfering with subsequent signaling induced by bacterial binding to the host cell [46]. Also, different nanobodies have been identified with potential application for immunodetection of cholera toxin and staphylococcal enterotoxin B [47], botulinum A neurotoxin complex [48], and toxic-shock syndrome [49].

Taken together, therapeutic mAbs can be exploited as targeting moieties for the development of drug delivery systems targeted towards infection-causing agents. More importantly, the therapeutic application of phage display-derived antibodies can be based on their immunological properties. This might occur through the capacity of antibody to recognize specific epitopes on the target pathogen that is followed by generation of a protective immune response or elimination of the pathogen. Antibodies can block the activity of infectious agents by neutralizing pathogen-derived toxins, triggering phagocytosis, and preventing pathogen attachment to the host cell. Disease-specific antibody libraries represent enormous potential for the production of monoclonal antibodies against a particular disease-causing agent. Raxibacumab [50] and palivizumab [51] are examples of PAL-derived antibodies that have exploited for for immunotherapy of human disease. These monoclonal antibodies might serve for the prophylaxis and treatment of *Bacillus anthracis* (the causative agent of anthrax) and respiratory syncytial virus bronchiolitis. Generally, PALs can speed up the process of generating desired antibodies against a specific antigen. This faster procedure of antibody production is of prime importance for emerging infectious diseases. In fact, PALs offer huge potential for the rapid development of therapeutically relevant antibodies against emerging infectious agents of high concern and genetically modified invading pathogens in an emergency condition. With the rise of new infectious diseases in particular newly-arisen viral infections, PALs can play a more important role in the establishment of efficient immunotherapeutics against these novel pathogens in the future. Since PAL-derived antibodies typically do not have common limitations of animal based antibodies, toxic agents (e.g. pathogen-derived toxins) and even live cultures of the disease do not pose barriers to the process of antibody generation.

## Bacteriophages as promising tools for vaccine development

The major goal of vaccination is to elicit a strong immune response that could lead to the formation and maintenance of a long-lasting immunity. Previously, vaccine research focused on developing prophylactic agents against pathogenic microbes. However, currently researchers also devote efforts to make vaccines against different disorders especially cancer. Many vaccine types are not able to initiate a powerful and effective immune response. This can largely be attributed to the absence of a proper delivery system for efficient activation of the immune system. Therefore, the development of optimal delivery systems is of particular importance for any type of vaccine. Bacteriophages have attracted attention of vaccinologists as useful vaccine delivery agents and as immunogenic carriers with potential application in the development of different vaccine types. The first use of recombinant phage as immunogen was described by de la Cruz et al. who cloned repeat regions of the circumsporozoite (CP) protein gene of *Plasmodium falciparum*, the causative parasite of malaria, into one of the minor coat protein genes of f1 filamentous phage [52]. They showed that recombinant phage preparations are both antigenic and immunogenic in rabbits. For application in vaccine design, phage particles should be able to deliver their cargo into target cells. For this reason, here we take a brief look at the capacity of phages for cargo delivery into eukaryotic cells in particular cells of the mammalian origin. Although bacteriophages are viruses that specifically infect bacterial cells, a range of convincing evidence strongly supports the idea that these prokaryotic viruses can also deliver their encoded genes into human cells. This was first discovered when Merril et al. in 1970 found the exposure of human fibroblast cells, derived from a child with congenital deficiency of the enzyme α-D-galactose-I-phosphate uridyl (GPU) transferase activity, to phages harboring a wild-type copy of transferase gene leads to the induction of the enzyme activity [53]. Within the recent years, a growing body of information has highlighted the importance of phages for gene transfer into mammalian cells. These findings have reinforced the notion that bacteriophages can be used as a novel and efficient category of gene delivery vehicles (GDVs) for the introduction of various diagnostic and therapeutic cargoes to human cells [12]. The development of such capacity in phages can be attributed to the long-standing and continuous evolutionary relationship between these viruses and the human body. Currently, it has been shown that phages, along with their bacterial hosts, form a major part of the natural microflora of the human body. In spite of the capacity of phage virions for the delivery of their payloads into mammalian cells, it is of interest to note that one should discriminate between the concepts of interaction and infection. This notion gains significance when it is observed that phages are able to interact with, but not infect and multiply in, mammalian cells. Formerly, it was though that phage DNA which is of a prokaryotic origin could not be functional in eukaryotes. However, it has now become evident that phage-derived genes can function properly in mammalin cells [54].

From a practical point of view, phage-based vaccines have been demonstrated not to cause any significant side effects in vivo [55]. Several lines of evidence in animals imply that high doses of the intravenously administered phages do not yield overt toxicity [56]. In fact, bacteriophages are not infectious for humans and could not act as pathogenic agents in the body. Authentically, they are much less likely to bind to the human cell surface receptors in a manner that trigger intracellular signal transduction pathways. As a result, downstream toxic effects inside the cells are inhibited. As phage particles are not able to propagate in eukaryotic cells, they behave as inert particulate entities and their application is considered much safer than other viral vaccines. In general, phages possess several characteristics making them well-matched for the development of vaccine delivery platforms. These viral particles are highly stable under a diverse range of harsh environmental conditions. They can be adapted inexpensively for large scale production by using simple bacteriological media and do not require cell culture systems to be manufactured in sufficient quantities [57].

Phages are intrinsically capable of stimulating both arms of the immune system (humoral and cell-mediated immunity). They have been demonstrated to be strong adjuvants and are able to boost immune response against any antigen which is presented together with the phage particle. This immunostimulatory effect of phage-based vaccines eliminates the need for adjuvants that are frequently administered along with recombinant proteins and synthetic peptides to improve immune response to vaccines. Thus, phages can be used as an alternative to classical carrier proteins. For example, f1 filamentous phage displaying the B2.1 peptide on it surface has been indicated to generate an immune response in mice more potent than coupling the peptide to traditional carriers such as ovalbumin [58]. One important factor contributing to the immunostimulatory property and adjuvant capacity of phages is the presence of a number of unmethylated CpG motifs in their genome. These motifs can account for the activation of the innate immune system through Toll-like receptors (TLRs) [59, 60]. The function of phages as a natural adjuvant abrogates the need for alternative adjuvant purification and further conjugation to the vaccine. Therefore, the incorporation of phage particles into the formulation of vaccine candidates enormously decreases the expense of producing new vaccines while increasing efficiency. Due to their particulate nature, phage vaccines can be taken up by antigen presenting cells (APCs). APCs engulf phages with antigenic and immunogenic determinants, process them efficiently, and present phage-mediated antigens through major histocomaptibility complex (MHC) class I and II pathways [61]. MHC-mediated peptide presentation stimulates both cellular and humoral immune responses. It has been revealed that phages as particulate antigens not only activate antibody production via MHC class II pathway, but also mount cell-mediated immunity through gaining access to MHC class I pathway.

Although vaccine administration through subcutaneous or intramuscular injection is more widespread than other administration routes, phage-based vaccines are stable in the gastrointestinal tract and thus represent potential to be used orally [62]. Neverthesless, there are some speculations on the risk of oral administration because phages can infect the gut microbiota and perturb the microbial flora of the body. To circumvent this problem, the exploitation of non-lytic phages might be a better strategy. Non-lytic phages can reduce the risk of destroying the natural gut bacteria. Consistent with this notion, phage vaccines have been demonstrated to produce strong immune response in mice when the animals are vaccinated through oral route. Several studies have confirmed the immunostimulatory effects of phage-based vaccines when used through oral administration [63–65].

## Immunological aspects of phages and phage-displayed peptides

Although the history of phage research is flooded with studies that address the interaction of phages with their bacterial hosts, it has been revealed that phage particles are also able to interact with different mammalian cells in particular cells of the immune system. These interactions mainly involve two areas: the first is focused on the immunogenicity of phage particles that is defined as the capacity of phages to elicit specific immune response. The production of anti-phage antibodies takes a prominent place in the issue of phage immunogenicity. The second area involves the immunomodulatory activities of phage particles that are linked to the phage nonspecific effects on the functions of immune cells implicated in both innate and adaptive immune responses.

### **Phages** and humoral immune response

The capacity of phiX174 phage for mounting in vivo humoral response has served for over thirty years in clinical immunology as an efficient tool to evaluate T helper cell-dependent antibody production in immunodeficient patients. There are different reports implying that phages are able to induce the production of specific antibodies. For example, it has been shown the enhanced levels of antibodies specific for staphylococcal phage can be found in > 40 % of individuals with bacterial infection that is significantly higher than about 10 % of non-infected healthy individuals. A significant decrease of the antibody titer is detected during infection regression [66]. Interestingly, the sera of non-immunized subjects may have a low level of anti-phage antibodies. The presence of these so-called natural antibodies are believed to result from the omnipresence of phages including different environments we are exposed to, food that we eat, and the normal microflora of our body [67]. Anti-phage antibodies belong to the category of neutralizing antibodies with capacity for binding to the viral epitopes that are essential for infecting host cells. Therefore, anti-phage antibodies can prevent phage from infecting their host bacterial cells. In general, the magnitude of the anti-phage humoral response appears to be dependent on different factors such as phage type, administration route, and the initial status of exposed individuals [68–70].

There are different studies highlighting that phage-displayed peptides are able to elicit specific anti-peptide humoral response. In this context, phages can be used as carriers for immunogenic epitopes obtained from infectious agents in order to raise antibody response specific for the displayed peptide. Consistent with this, two 12-mer peptide epitopes of the circumsporozoite protein of *Plasmodium falciparum* called MAL1 and MAL2 displayed on filamentous phage have been able to induce a specific IgG antibody response in mouse models [71]. Further investigations showed that this immune response is T cell-dependent. Also, fd phage-displayed peptide epitopes from the V3 loop of the surface glycoprotein gp120 [72], reverse transcriptase of the human immunodeficiency virus (HIV) [73], and glycoprotein G of the human respiratory syncytial virus (RSV) [74] have been capable of inducing the production of high titers of pathogen-specific antibodies and conferring resistance to the infectious agent on vaccinated animals. Taken together, these observations provide strong support for the notion that peptide epitopes displayed on the surface of phage particles represent huge potential to be used as efficient means for focusing the antibody response to the pathogen-derived epitopes and triggering a potent pathogen-specific humoral response.

### Phages and T cell-mediated immune response

T cells are known to play important roles in the effective clearance of virus-infected cells and the inhibition of growth and expansion of tumor cells. This can occur via both CD8^+^ and CD4^+^ T cells that provide the cellular arm of adaptive immunity against pathogenic viruses. The capacity of activating a potent cytotoxic T lymphocyte (CTL) response is of utmost importance for defense against viral infections and is assumed to be an important feature needing to be considered in the development of vaccine platforms. One of the main limitations of vaccines that contain non-living components is their lack of ability to reach the MHC I pathway and activate CTL response. Although phage-mediated T cell-dependent immunity has been studied less than anti-phage humoral response, there are numerous lines of evidence indicating that phage-displayed peptide epitopes can mount efficient cellular immune responses [61]. It has been revealed that phage particles provide the means by which exogenous antigens can reach the MHC class I pathway. In fact, phages displaying epitopes - both from tumor associated antigens (TAAs) and from viral peptides – are able to present the epitopes in a highly immunogenic form and elicit strong peptide-specific CTL responses both in vitro and in vivo [75]. In this context, display of the peptide RT2 derived from the reverse transcriptase of HIV-1 on fd phage has led to the priming of an epitope-specific CTL response in human cell lines. Also, mice immunization with this epitope-displaying phage has resulted in an efficient and specific CTL response against HIV-RT2 [76].

### **Phages**, innate immunity and inflammation

Innate immunity refers to nonspecific reactions of the body to foreign antigens. In this procedure, universal pathogen-associated molecular patterns (PAMPs) are recognized by different pattern recognition receptors (PRRs) expressed by most defense cells of the host. PRRs provide an immediate response against the invading pathogen. Also, there are a variety of soluble PRRs in the blood that act as opsonins and trigger the complement pathways [77]. TLRs constitute one of the best-characterized and most important classes of PRRs. These receptors are expressed by phagocytic cells and play significant roles in the innate immune response including inflammatory reactions. PAMP recognition by PRRs activates cells that release signaling molecules such as cytokines. These cytokines act as messengers and on their own stimulate the activity of other elements of the immune system. The activation of innate immunity is related to inflammatory reactions that occur in response to the presence of foreign antigen in the body [78]. It has been revealed that PAMP recognition and subsequent TLR-mediated activation of monocytes and macrophages lead to the elevated production of reactive oxygen species (ROS), nitric oxide, and a variety of inflammation-associated cytokines such as interleukin-12 (IL-12) and interleukin-23 (IL-23).

### Phages and cytokine production

There are a variety of studies that indicate phages can have substantial impacts on the production of various cytokines. These effects can be on the expression levels of both pro-inflammatory and anti-inflammatory cytokines. The injection of T7 and fd phages has been found to increase interferon gamma (INF-γ) levels [79]. The purified preparations of *Staphylococcus aureus* phage are able to induce the production of IL-6 and the purified T4 phage preparations inhibit the production of mitogen-induced IL-2 [68]. Data gathered from experimental phage therapy on animals have exhibited that successful therapy corrects the enhanced levels of pro-inflammatory cytokines related to bacterial infections [80]. Also, treatment of burn wound infection caused by *Klebsiellapneumoniae* has been associated with reduced levels of IL-1β, tumor necrosis factor alpha (TNF-α), and IL-10 in the sera and lungs of phage-administered mice [81]. The major part of information about the effects of phages on regulating the levels of different cytokines has been obtained from phage therapy studies. On the other hand, there are many unmethylated CpG motifs in the genome of phages that can be recognized by TLR9. This elicits a Th1-dominated response leading to the release of numerous cytokines such as INF-γ, TNF-α, IL-2, IL-6, and IL-18 [82]. It has been revealed that cytokine production depends on several factors. Importantly, phage type dictates the type and the level of cytokines that are produced. Also, phage therapy studies have confirmed that cytokine production depends on the initial responsiveness of individuals. For example, patients with a low or moderate TNF-α levels in the serum upregulate this cytokine, but patients with initially high serum levels of TNF-α downregulate this cytokine following phage therapy [68].

### Phages and connecting innate immunity to adaptive immunity

Phages apply different routes to link together specific and nonspecific immune responses, thereby connecting innate component of the immune system to adaptive immunity. Phage recognition by anti-phage antibodies triggers the activation of serum complement pathway. Complement activation contributes to the activity of reticuloendothelial system (RES). Phages circulating in the blood may be inactivated by the RES macrophages. RES and anti-phage antibodies are involved in the clearance of phage particles from the body. One of the outcomes of complement activation is the production of complement cleavage products that interact with diverse receptors found on the surface of myeloid, lymphoid, and stromal cells. This interaction provides a basis for the regulation of both B and T cell-mediated immune responses [83]. Also, CpG motifs found in the phage genome are also capable of connecting the innate and adaptive immune responses through their capacity for activating antigen-presenting cells (APCs).

## Phage display vaccines: bacterial viruses en route to antigen delivery

**Fig. 3:**
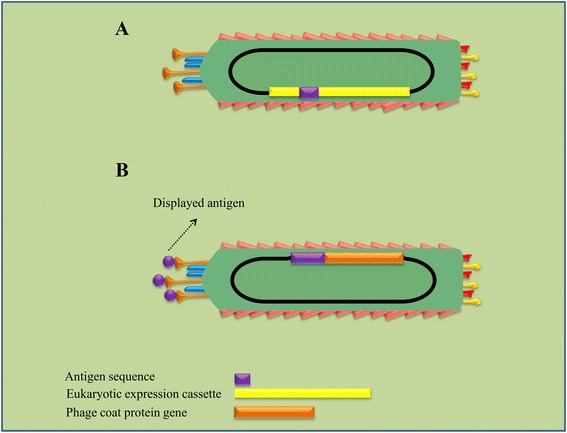
Phage DNA vs. phage display vaccine a) In phage DNA vaccine, a eukaryotic gene expression cassette containing the antigen sequence is cloned into the phage genome. Therefore, the antigen expression is under the control of eukaryote-derived regulatory sequences. b In phage display vaccine as the main phage-based strategy for vaccine design, the vaccine sequence is cloned into the phage coat protein gene. Therefore, the antigenic amino acids are expressed as fusion to the coat protein on the phage surface. These antigenic amino acid residues are readily presented to the immune system

The exploitation of phages as vaccine delivery vehicles can be divided into two main categories including phage DNA vaccine and phage display vaccine (Fig. 3). In phage DNA vaccine, the antigen-coding gene which has been cloned into a eukaryotic cassette is inserted into the phage genome. The eukaryotic cassette contains sequences essential for the proper regulation of gene expression including promoter, 5′-UTR (5′- untranslated region), ORF (open reading frame), 3′-UTR (3′-untranslated region), and poly (A) tail. Therefore, the antigen-coding gene is controlled by a strong promoter and efficient regulatory sequences. After amplification in bacterial cells, the whole phage particles are purified and then injected into the host as an immunization tool. Unlike standard DNA vaccines, here the vaccine-producing sequence is protected from degradation by the phage coat proteins. Therefore, these vaccines have higher stability for administration (in particular as oral vaccines), storage, and transport. Compared with standard plasmid DNA vaccination, phage DNA vaccines have been demonstrated to yield better results at lower doses. These vaccines have been used to immunize different small and large animal models and the results have demonstrated that they can induce a significantly higher and longer-lasting antibody response in comparison with naked DNA vaccine or even purified recombinant proteins [84–86]. However, the most important contribution of phages to vaccine development is the production of phage display vaccines. This phage-based antigen delivery approach is gaining a growing interest and has been suggested as a prospective tool in the area of vaccine design. Within the past years, phage display vaccines have represented a promising approach for vaccine development. In phage display vaccine, the foreign antigen is displayed on the phage surface. This is generally achieved by expressing the antigen of interest as fusion to one of the surface proteins of the virion. Since there are multiple copies of the desired antigen on the surface of each phage particle, phage-displayed vaccines are able to provide a high level of antigen exposure to the immune system. However, the display of antigenic epitopes in a properly folded and functioning conformation that could elicit a strong immune response is a significant issue which deserves further attention in the development of phage display vaccines. Sometimes, rather than constructing a transcriptional fusion to a coat protein, phage disply vaccines may be created by conjugating the antigenic substance to the phage surface via artificial linkers [87]. The exploitation of this strategy for the construction of phage display vaccines may diversify the range of antigens that can be displayed on the surface of immunogenic phage particles. However, antigen is not encoded by the phage genome in this strategy and thus the vaccine construct should be prepared every time it is needed. This is in striking contrast to the genetic approach in which phage virions have the antigenic sequence in their genomes. Therefre, they can continually produce the vaccine construct through convenient bacterial amplification and do not need to repetitively attach the antigenic substance to the phage surface.

Different phages have been used for the display of antigens in phage display vaccine platform including M13, lambda, T4, T7 and even some RNA phages. M13 has been applied as a popular platform for the development of vaccines against different pathologies including cancer and a variety of parasitic and bacterial infections. The body of this filamentous phage is covered by 2700 copies of the major coat protein (pVIII or P8) and is capped at both ends with five copies of each of the minor coat proteins including pIII (P3), pVI (P6), pVII (P7), and pIX (P9) [88]. Lambda phage is another important system for the development of phage display vaccines. Foreign antigens are fused to gpD. There exist 450 copies of this protein on the phage surface. The ability to display a high copy number of the same antigen on the surface of an individual phage virion is highly effective in triggering an appropriate immune response compared with displayed antigens of low copy number. The fusion product of antigen-gpD has been shown to generate neutralizing antibodies against porcine circovirus infection [89]. T4 is another bacteriophage which has represented potential as a vaccine platform. This phage has two highly immunogenic proteins on its outer surface, called Soc and Hoc [90]. These coat proteins can effectively serve to display foreign antigens on the phage surface and have domains that ideally expose the fused epitopic structures to the immune system. T4 is more adapted for the display of peptides with various sizes. To display antigenic substances on T7 phage, antigens are commonly fused to the carboxyl end of the capsid protein 10B. The display of human-derived vascular endothelial growth factor (VEGF) on the capsid of T7 phage has been demonstrated to produce strong immune response against cancer cells and inhibited the growth of cancer cells when injected into mic with Lewis lung cancer xenograft tumors [91]. Bacteriophages with RNA genomes have also been applied to develop vaccines. QB and MS2 are RNA viruses that antigens fused to their surface coat proteins have been used for the production of vaccines against drug addiction [92, 93] and HIV infection [94].

## Filamentous phages: popular systems for vaccine development

Filamentous phages constitute a group of viruses that are able to infect gram-negative bacteria with F plasmid. These viruses belong to the Inoviridae family and the genus Inovirus. Filamentous phages of the Ff class including M13, fd, and f1 are generally the most predominant phage display vectors. Accordingly, they are the most popular systems for the generation of phage display vaccines. These rod-shaped bacterial viruses have the simplest capsids in terms of the number of different protein types and harbor a single-stranded DNA genome that is ideal for genetic engineering purposes. Filamentous phages are as immunogenic as traditional carrier proteins such as bovine serum albumin (BSA) and keyhole limpet hemocyanin (KLH) [95, 96]. However, they contain relatively few endogenous B cell epitopes whose presence may divert the antibody response from its intended target. This might be ascribed to the connection between filamentous phages and the human body during a long evolutionary period. These phages infect *Escherichia coli* which is a commensal bacterium residing in the human gut. To be capable of living in the human body, filamentous phages have evolved proteins that induce a low-level antibody response. In fact, they apply this mechanishm to evade the mucosally generated antibody responses. For example, pIII is the only coat protein of filamentous phages that is large enough (approximately 400 amino acids) to present a variety of B cell and T cell epitopes [97].

Typically, peptides displayed on the surface of phage particles have been demonstrated to elicit antibody production in experimental animal models. However, this depends on the characteristics of the displayed peptide and the method of its display. In general, pIII and pVIII are known as the most widely used coat proteins of filamentous phages for the display of exogenous amino acid sequences. Due to its high copy number, pVIII is well-matched for the display of peptides and small proteins and cannot be considered as an efficient platform for the display of large antigens. By contrast, pIII has a low copy number and can be exploited effectively to display large antigens. This implies that the copy number of the displayed antigen is a critical factor requiring much consideration in vaccine development. Dispalyed peptides with a low copy number (pIII fusions) tend to generate a lower immunogenicity than displayed peptides with a high copy number (pVIII fusions). The low copy number of antigens displayed as fusion to pIII may pose a barrier for phage display vaccines and turns these vaccine types into a suboptimal choice. However, pVIII provides the advantage of high valency display of foreign antigens on the phage surface. In line with this, peptide-pVIII fusions containing mimotopes of human hepatitis B virus surface antigen (HBsAg) have been indicated to produce more potent antibody responses in mice and rabbits compared with phage-displayed mimotopes as fusion to pIII, fusion to human H ferritin, fusion to hepatitis B virus (HBV) core peptide, and synthesized multiple antigenic peptides carrying the amino acid sequence of the mimotopes [98]. Generally, the copy number increase of displayed peptides is made possible when recombinant fusion proteins are efficiently accommodated or packed on the phage surface.

## Phage library screening and mimotope identification

It has been revealed that peptides are able to effectively mimic the epitope structures present in antigens. These peptides are called mimotopes. One of the most promising applications of phage display technology is to pan RPLs against a specified target for the identification of mimotopes. As mimotopes mimic the structure of antigenic epitopes, they offer potential to be used as vaccine candidates. Based on this, vaccine researchers can screen RPLs for the selection of immunogenic mimics that are capable of eliciting powerful antibody responses in vivo. When peptide sequences potentially mimicking the conformation of an antigenic epitope are successfully isolated through library screening, they require further analyses so that their immunogenic and antigenic properties can be determined. If a mimotope derived from the library is known as a potent immunogen and antigen, it can be considered as a suitable candidate for vaccine production.

Mimotopes should undergo a pre-validation step. In this procedure, these mimotopes are evaluated by bioinformatic analyses and experimental assays such as ELISA and subsequently the best selected phage clones (e.g. with the highest reactivity values in ELISA assays) are chosen for further investigations. For ex vivo and in vivo assessments, either the selected phage clone or the synthetic mimotope can be used directly for animal immunization. When using phage clones as immunogen, the wild-type M13 phage may be exploited as control because phage particles on their own harbor proteins that can interact with the host immune system resulting in the development of a nonspecific immune response. This nonspecific response can interfere with specific immune response induced by the phage-displayed mimotope. When using the selected mimotope for animal-based immunological tests, one helpful strategy is taking advantage of the repeated peptide sequences separated by spacers that aid in appropriate conformational arrangement of the original epitope structure. Repeating the selected immunological motif makes it possible to mimic the phage capsid structure of the pIII protein. Sometimes BSA can be conjugated to the peptide sequence to raise the immune response. After immunizing animals with either the phage clone (with displayed mimotope) or the synthetic mimotope, the animals are challenged by infection with the target pathogen. The infected animals are monitored and those surviving several weeks (usually 10 weeks) post-challenge are sacrificed, their blood and spleen are collected, and used for analyzing the humoral and cellular responses [99]. The humoral immune response is assessed by measuring the IgG isotype distribution including IgG, IgG1 and IgG2a in the sera of immunized animals. The elevated production of IgG2 and IgG1 may be considered as an evidence of Th1 and Th2 immune response, respectively. To investigate the cellular immune response, splenocytes and peripheral blood mononuclear cells (PBMCs) are cultured and the production levels of a variety of cytokines are determined in the culture supernatants. Different interleukins (IL-4, IL-6, IL-10, IL-12), IFN-γ, IFN-c, TNF-α, and granulocyte macrophage colony-stimulating factor (GM-CSF) are some molecules commonly used for cytokine production analysis. Enzyme-linked immunospot (ELISpot) assay is known as a widely exploited methodology for monitoring the cell-mediated immunity. This technique can be adopted for the detection and quantification of cytokine-secreting cells [100]. Another approach that may be used to examine the protective efficacy of the mimotope is monitoring the parasite burden in the liver, spleen, bone marrow, and lymph nodes of the immunized animals within a several-week period post-infection [101]. Significant reductions of the pathogen load in these tissues may present the efficacy of the selected mimotope as an efficient vaccine.

One of the advantages provided by phage display library biopanning in mimotope identification for vaccine production is the ability to screen RPLs against polysaccharide-specific antibodies. Therefore, this methodology might be used to obtain peptidomimetics of polysaccharides. This selection scenario is of particuar importance for the production of novel vaccines against encapsulated bacteria. The polysaccharide capsules of these bacteria often possess antigenic features. In this setting, RPLs have been exploited for the isolation of peptide mimics of the polysaccharide capsule of *Neisseria meningitidis* including serotypes A [102], B [103], and C [104] of this pathogenic bacterium. The mimotopes identified against these serotypes have been able to mount protective antibody responses in murine models. Phage display methodology can be exploited in another context to achieve vaccine candidates. Phage display libraries may be screened against a specific antiserum to recognize novel mimotopes. In this situation, the immune serum of convalescent individuals serves as the target of biopanning and thus potential vaccine candidates against a specific disease are isolated. This procedure can be performed without prior knowledge of the characteristics of protective antigen. In this regard, the screening of RPLs on serum derived from individuals with HIV-1 [105] and HBV infections [106] has led to the identification of peptidomimetics of some viral epitopes. The immunization of animal models with these peptide mimics has resulted in the production of virus-neutralizing antibodies. This approach has also been used against serum samples from cystic fibrosis (CF) patients with *Pseudomonas aeruginosa* infections and led to the identification of several antigens which encode outer membrane proteins and secreted proteins of the bacterium [107]. These antigens are suggested to be potential targets for vaccine generation against *Pseudomonas aeruginosa* infections. To increase the chance of isolating phage-displayed peptides with high affinity binding to disease-specific antibodies, the phage library can be first exposed to negative screening on sera from non-infected people followed by positive selection on sera from infected patients. This strategy has been used for the isolation of mimotopes against the parasite *Brugia malayi* (one of the causative agents of lymphatic filariasis) [108]. Of several identified antigens, one conferred protection to the parasite when animals were challenged with the infection.

A wide variety of pathogens have been used as the target in phage display selection through biopanning. Thus far, the screening of phage display libraries has been applied to identify peptidomimetic vaccine candidates against numerous disease-causing infectious agents. Table 1 lists important studies in which phage display biopanning has served to identify mimotopes for numerous pathogenic organisms. These mimotopes represent potential application in vaccine formulation for preventing diseases caused by the pathogens.

**Table 1:** screening of phage combinatorial peptide libraries against different pathogens for mimotope identification

Infectious agent	Pathogen type	Reference
Anaplasma marginale	Bacterium	[109]
Avibacterium paraglinarum	Bacterium	[110]
Brucella sp.	Bacterium	[111]
Entamoeba histolytica	Ptotozoa	[112]
Fasciola hepatica	Flatworm	[113]
Hepatitis A virus (HAV)	Virus	[114]
Hepatitis B virus (HBV)	Virus	[115]
Hepatitis C virus (HCV)	Virus	[116]
Herpes simplex virus - 2 (HSV-2)	Virus	[117]
Human immunodeficiency virus - 1 (HIV-1)	Virus	[118] [119]
Ixodes scapularis	Tick	[120]
Japanese encephalitis virus	Virus	[121]
Leishmania infantum	Protozoa	[101] [122] [123] [124]
Leishmania major	Protozoa	[125]
Leptospira interrogans	Bacterium	[126]
Mycoplasma hyopneumoniae	Bacterium	[127]
Niaph virus	Virus	[128]
Plasmodium falciparum	Protozoa	[129]
Rhipicephalus microplus	Tick	[130]
Schistosoma japonicum	Flatworm	[131] [132]
Streptococcus pneumoniae	Bacterium	[133]
Strongyloides stercoralis	Roundworm	[134]
Taenia solium	Tapeworm	[135] [136]
Toxoplasma gondii	Protozoa	[137]
Transmissible gastroenteritis coronavirus (TGEV)	Virus	[138]
Trichinella spiralis	Roundworm	[139] [140]
Trypanosoma cruzi	Protozoa	[141]

## Conclusion and future perspective

Although bacteriophages phylogenetically fall into the category of viruses, they present a picture completely different from the portrait we have from viruses. Phages are not infections agents for eukaryotes and therefore could not trigger pathogenesis in the human body. Indeed, they can even be exploited as useful agents for prophylactic and therapeutic purposes. Phage-based vaccines not only serve as preventative platforms for battle with microbial and parasitic infections, but also can find utility as therapeutic platforms for combat against non-infectious diseases. The therapeutic application of phage-based vaccines is achieved through immunotherapy that is based on harnessing the innate power of body’s natural defense to fight the disease. In this manner, phage-inspired vaccines can be directed towards treatment of cancer, neurodegenerative disorders, drug addiction, etc. A body of convincing evidence reinforces the notion that phages are able to interact with and release their cargo inside mammalian cells, thus being suggested as a novel gene/drug delivery vehicle. However, as phages are specific antagonists to prokaryotic hosts, their natural capacity for eukaryotic cargo delivery remains largely limited. The surface engineering of phages through phage display can dramatically improve the efficacy of these viral particles for the delivery of prophylactic, diagnostic, and therapeutic cargoes into eukaryotic hosts. This strategy alters surface binding properties and consequently the host range of phage virions via capacitating them for interacting with mammalian cells.

Phage display is a technology that has made a revolution in biomedical applications of bacteriophages. Actually, the birth of this versatile methodology has been an important milestone in the history of phage research and contributed tremendously to the field of vaccine development. This innovative technique brings the opportunity for expressing a wide variety of antigens on the surface of phage particles. Accordingly, phages might be adopted as vaccine delivery vehicles. In all phage display systems by using different phages as well as different proteins of a certain phage, antigens are fused to the phage coat proteins. Since several copies of the coat protein are present on the phage surface (sometimes up to several hundred or thousand copies), each virion carries multiple copies of the antigen of interest on its surface. As a result, a large amount of antigen is exposed to the body’s immune system. But, one of the most relevant contributory factors in the maturation of phages into optimal vaccine delivery systems is their imunostimulatory characteristics and potent adjuvant capacities. These critically significant attributes turn phages into efficient immunogenic carriers which can mount both humoral and cell-mediated immunity. Compared with other viral groups, phages interestingly offer a far higher degree of in vivo safety making them well-matched tools for translation from bench to bedside. Also, the rise of combinatorial peptide libraries has been a shining star in the firmament of phage applications. These randomized libraries with a rich collection of peptide ligands have transformed phage display into a high throughput screening approach with huge potential for the selection of specific ligands. The existence of an exhaustible reservoir of peptides enables vaccine researchers to find potential mimotopes with high antigenicity and immunogenicity in phage display libraries. These rare functional mimotopes among a large population of nonfunctional peptides are assumed to be gems in the junk whose capacity for mimicking the antigenic structures can be used for immunostimulation. Another contribution of phage display to the field of vaccine production is its potential for the development of hybrid phage vaccines. These vaccine types are constructed by combining the two approaches of phage DNA vaccine and phage display vaccine. In this combinatorial platform, phage particles carry a foreign antigen-encoding gene in their genome and at the same time display a peptide or protein on their surface. The displayed peptide or protein may be a targeting molecule. For example, it can be selected through biopanning for its specific binding affinity towards APCs such as dendritic cells. Accordingly, hybrid phage vaccines can directly deliver the antigen-encoding sequence to immunoreactive cells.

Affinity maturation can be performed through several rounds of library generation/mutagenesis and selection [142, 143]. The selective enrichment of high-affinity antigen-specific ligands from second-generation libraries (SGLs) can be implemented by screening with lower concentration of the target antigen, shorter incubation times, longer or more extensive washing steps, and display of mutagenized molecules at a lower valency on phage particles. the establishment of SGLs relies on introducing mutations into the nucleotide sequence encoding the originally selected binder. Several methodologies are available for the purpose of mutagenesis. These methods generally fall into two categories of site-specific and random mutagenesis. Site-specific mutagenesis is performed through site-directed mutagenesis and cassette mutagenesis and random mutagenesis is achieved through error-prone PCR and DNA shuffling [142, 144]. Also, next-generation sequencing (NGS) is recently gaining popularity in phage display. The traditional approach of phage display screening relies on multiple rounds of affinity selection that are followed by standard Sanger sequencing. Sanger sequencing is labor-intensive and low-throughput resulting in sequencing of inserts from a very limited number of phage clones (representing only < 0.01 % of the complete sequence space of the library) [145, 146]. Also, repeated rounds of selection might lead this small sample space to be dominated by nonspecific binding sequences with faster amplification rates known as parasitic sequences [147] or with binding capacity to the components of the screening system in particular plastic surface of the solid support [148]. In this context, the final outcome is the isolation of false positive hits with no actual specific binding to the target at the end of biopanning. NGS provides the possibility to characterize >10^6^ sequencing reads in a single run. Furthermore, this high-throughput characterization of the diversity of binding variants can be performed in earlier rounds of biopanning (after 1–2 rounds of selection on the desired target) [149]. Amplifications that are performed between selection rounds enhance the risk of introducing biological bias and collapse of the library diversity. By reducing the number of amplification rounds, the incorporation of NGS platforms into phage display screening decreases the chance of isolating nonspecific false positive clones and accelerates the process of target-specific ligand identification.

Beyond doubt, phage display cannot bypass all barriers in the way of vaccine production and is not able to resolve all problems we might face in our efforts to generate ideal vaccines. However, it is crystal-clear that this powerful methodology can offer a wide variety of appropriate keys to unlock the doors ahead. Current situation promises a bright future for different phage-based vaccine platforms and it can be argued that the role of phage display will continue to grow in the coming years. With ongoing progress being made in the exploitation of phages for epitope mapping and antigen presentation, the research community hopes to find new tactics and strategies for vaccine discovery and development.

## Abbreviations

APC: Antigen presenting cells
APD: Antibody phage display
BSA: Bovine serum albumin
CDR: Complementarity determining region
CF: Cystic fibrosis
CP: Circumsporozoite
CTL response: Cytotoxic T lymphocyte response
ELISA: Enzyme-linked immunosorbent assay
ELISpot: Enzyme-linked immunospot
Fab: Fragment antigen binding
GDVs: Gene delivery vehicles
GM-CSF: Granulocyte macrophage colony-stimulating factor
GPU Transferase: α-D-galactose-I-phosphate uridyl (GPU) transferase
GST: Glutathione S-transferase
HBS: Hepatitis B virus
HCAb: Heavy chain antibody
HIV: Human immunodeficiency virus
HuCAL: Human combinatorial antibody library
ICC: Immunocytochemistry
IHC: Immunohystochemistry
IL: Interleukin
INF-γ: Interferon gamma
KLH: Keyhole limpet hemocyanin
LPS: Lipopolysaccharide
mAb: Monoclonal antibody
MBP: Maltose-binding protein
MHC: Major histocompatibility complex
MOPS: Magnetic beads via organic phase separation
NGS: Next-generation sequencing
NPLs: Natural peptide libraries
PAL: Phage antibody library
PAMPs: Pathogen-associated molecular patterns
PBMC: Peripheral blood monuclear cell
PRRs: Pattern recognition receptors
RES: Reticuloendothelial system
ROS: Reactive oxygen species
RPLs: Random peptide libraries
RSV: Respiratory syncytial virus
scFv: Single-chain variable fragment
SCID: Severe combined immune deficiency
sdAb: Single domain antibody
SGL: Second-generation library
TAA: Tumor associated antigen
TLRs: Toll-like receptor
TNF-α: Tumor necrosis factor alpha
UTR: Untranslated region
VEGF: Vascular endothelial growth factor
VH: Variable region of heavy chain
VHH: Variable domain of HCAb
VL: Variable region of light chain
VSG: Variant surface glycoprotein
